# Assortative Mating for Emotional Intelligence

**DOI:** 10.1007/s12144-016-9501-8

**Published:** 2016-09-05

**Authors:** Magdalena Śmieja, Maciej Stolarski

**Affiliations:** 1 Institute of Psychology, Jagiellonian University, Krakow, Ingardena Str. 6, 30-060, Krakow, Poland; 2 Faculty of Psychology, University of Warsaw, Warsaw, Poland

**Keywords:** Emotional intelligence, Assortative mating, Couples, Dyads, Initial assortment

## Abstract

Assortative mating has been studied on a broad range of variables, including intelligence and personality traits. In the present study we analysed the effect of assortative mating for ability emotional intelligence (EI) on a sample of heterosexual couples (*N* = 382), including dating and married couples. Correlation analyses revealed moderate similarity of Pearson’s *r* = .27 for general EI score, and was slightly weaker (from .18 to .23) for branch scores. Regression analyses showed that the Perception branch was the strongest single predictor of a partner’s general EI score, both in males and females. Continuous parameter estimation (CPEM) revealed that the magnitude of the correlation does not increase with age, thus it is highly possible that the obtained similarity reflects initial assortment (i.e., similarity at the starting point of the relationship), rather than convergence (i.e., increasing similarity with time). It seems that EI is a significant factor influencing mate assortment processes.

## Introduction

While not many lay theories find their confirmation in psychological science, the old saying “Birds of a feather flock together” is one of those truly proved. Many studies show that people tend to be similar to their long-term partners, and this congruence is associated with higher relationship longevity (Rammstedt et al. 2013), and higher relationship satisfaction (Gonzaga et al. 2010). Such intra-couple resemblance, much larger than if it happened through random choice, implies systematic selection of partners based on similarity. This phenomenon labelled as assortative mating (Buss 1984, 2003) has been widely investigated, both within the scope of biology (e.g., Dieckmann and Doebeli 1999), and psychology (e.g., Luo and Klohnen 2005). Positive assortative mating (similarity; also labelled as ‘homogamic mating’) is indicated by a positive correlation between male and female partners’ scores on the same characteristic, while negative assortative mating (complementarity; heterogamic mating) is indicated by those scores being correlated negatively (Watson et al. 2004). Although it is possible that couples may ‘converge‘, and become more alike in personality over time (convergence: Gonzaga et al. 2010), partner selection is considered the more important factor in assortative mating (initial assortment: Keller et al. 1996).

The phenomenon of assortative mating is widespread across different species, and with respect to various characteristics. It may occur at the level of particular traits, and at the level of the overall mate value. Systematically, some variables consistently yield much stronger similarity correlations than others (Keller et al. 1996; Watson et al. 2004). The strongest positive assortment is found for sociodemographic variables and attitudes (Buss 1984; Escorial and Martín-Buro 2012; Feng and Baker 1994). For example, correlations between spouses for age range from .70 to .90 (Buss 1985), and for the social class of the neighbourhoods where they lived before marriage reach .71 (Hollingshead 1950). Assortative pairing for intelligence, education, and cognitive abilities is lower, but still substantial, ranging from *r* = .33 to *r* = .55 (Colom et al. 2002; van Leeuwen et al. 2008). For personality, findings are mixed and estimates vary across studies (e.g., Gonzaga et al. 2010; Luo and Klohnen 2005). Some authors report a low degree of similarity for personality traits in couples (Watson et al. 2004), some suggest it is moderate (Escorial and Martín-Buro 2012), while others reveal correlations above the level of .40 (McCrae et al. 2008), which is similar to the strength of relationships obtained for intelligence (see meta-analysis by Bouchard and McGue 1981).

Surprisingly, a very small number of studies have been dedicated to assortative pairing for such an important factor of partner selection and relationship satisfaction as emotional intelligence (EI). As defined by Mayer and Salovey (1997), emotional intelligence includes abilities to perceive, use, understand, and manage emotion. All of these seem fundamental to human mating and close relationships. An accurate perception of emotions enables correct interpretation of nonverbal emotion cues conveyed by the partner, which significantly improves relationship satisfaction (Feeney 1999; Gottman and Porterfield 1981), and security (Cordova et al. 2005). High efficiency in detecting incoherent affective expression characteristic for emotional deception (Wojciechowski et al. 2014) allows emotionally intelligent persons to identify their partner’s concealed problems and react in an appropriate way at the very beginning of a problematic situation. High ability of perceiving emotions helps in recognizing early signs of dissatisfaction or sadness, and subsequently – prevent their escalation. Using emotions to facilitate thinking may ease emphatic concern for the partner. Understanding one’s own emotions might be a valuable source of information about the current condition of the relationship. This ability may also help to articulate feelings and needs, predict partner’s emotional states, or even forgive partner’s transgressions (Fitness 2001). Higher understanding of emotions may reduce the severity of disagreements by helping to understand how the other person’s decisions are influenced by his or her current affective state. This ability can also help to foresee the dynamics of one’s own emotions, and thereby avoid destructive scenarios for conflicts. Effective emotion management might inhibit aggressive or destructive behaviours and promote positive reactions. It can be useful for maintaining emotional well being of both partners during conflicts. Research suggests that emotionally intelligent individuals tend to engage in active and constructive strategies of conflict resolution, avoiding those characterized as passive and destructive (Stolarski et al. 2011).

The benefits of romantic partner’s emotional skills seem crucial from the evolutionary perspective as well. A satisfying relationship with an emotionally intelligent partner adds to physical and psychological well-being, and through those protects from internal and external risks, thereby increasing chances for survival. As some heritable mental disorders (e.g., depression, anxiety disorders, schizophrenia) reduce the capability for accurate perceiving, understanding and managing emotions, high emotional intelligence might be construed as a symptom of mental health, and an indicator of good genetic quality.

The evolutionary benefits of emotional intelligence may differ for men and women. According to Triver’s theory of parental investment (Trivers 1972), mate selection is driven by different levels of contribution made by males and females for their offspring. As female parental investments of time and energy dedicated to the child are usually much bigger than the male’s, women place greater importance on finding a partner who is trustworthy, capable of acquiring resources, and willing to invest them into the current relationship. From that point of view, emotionally intelligent men might be especially attractive, because they can be expected to provide a larger-than-average amount of resources, as having superior leadership skills (Rosete and Ciarrochi 2005), and showing improved work performance (Côté and Miners 2006), which may translate into earning capacity. Empathy and advanced social competences (Lopes et al. 2004; Brackett et al. 2006a, 2006b) associated with high emotional intelligence may help in developing family bonds and creating stable, supporting environment for the growing child. On the other side, low EI of the father, associated with aggressive, destructive or illegal behaviours (Brackett et al. 2004; Trinidad and Johnson 2002) may decrease the chances of offspring survival, or at least – of their well-being.

According to Trivers’ theory (Trivers 1972), male parental investments are usually much smaller than the female’s. Although they might cover providing material resources, defending the family against aggressors, transferring knowledge, status and power to the child, sometimes they are minimal and limited to copulation. Regardless of the amount of resources dedicated to the child, the strategic aim for the male is finding a partner who is both able to get pregnant and to take good care of the baby. From that perspective, high EI female partners are particularly attractive because of being better in parenting as more emphatic, supporting mothers, and competent coaches of the child’s social development.

The utility of emotional abilities described here depends on the mating strategies employed by a man or a woman. Emotional skills seem very important in long-term relationships, which involve the need for efficient cooperation, solving everyday problems, and managing conflicts. Since people, especially women, seek warm, kind and altruistic romantic partners for long-term relationships (Buss 1989, 2003; Barclay 2010; Farrelly et al. 2016), for those who are ready to develop that kind of attachment, EI should be more valued and wanted. In short-term relationships, as those excluding enduring close interpersonal contact, EI could make an advantageous trait only as far as it is perceived as a desirable heritable characteristic.

For all of the reasons listed above, people should look for the most emotionally intelligent partners possible. Indeed, there is empirical evidence showing that EI is one of the most desirable traits of an ideal partner (Amitay and Mongrain 2007; Schutte et al. 2001). However, because human mating decisions take place in a dynamic mating market characterized by mutual mate choice, not everyone can choose the partner representing the highest level of desirable traits, and most people face a trade-off between their preferences and their own resources. As a result, people tend to mate with partners whose emotional abilities are similar to their own (Dillon et al. 2015). Therefore, would positive assortment be detectable in the case of emotional intelligence? So far, the evidence on assortative mating for EI is scarce and mixed. Some authors (e.g., Brackett et al. 2005; Zeidner and Kaluda 2008; Zeidner and Kloda 2013) reported no significant correlation between both partners’ EI, whereas others did (e.g., Brackett et al. 2006a, 2006b; Stolarski et al. 2011). Regrettably, the sample size in all of those previous studies was rather small and participants were mostly involved in short-term relationships. Moreover, the issue of assortative mating was rather a side result than a central, deeply analysed or exhaustively discussed effect of the investigations. The current study was designed to address those limitations.

### Aims and Hypotheses

The aim of the present study was to verify whether the assortative mating effect exists for EI, as well as to determine its size. In Mayer and Salovey’s (1997) theoretical model, EI is operationalized as a set of cognitive abilities used for processing emotionally relevant information. According to these authors, EI meets traditional standards for intelligence and could be treated as a kind of standard intelligence (Mayer et al. 1999). Therefore, we expected a positive assortment on the general EI level, similar to estimation levels found for general intelligence.

## Method

### Participants and Procedure

A total of 382 heterosexual couples participated in this study (764 participants). The sample consisted of university students and graduates. Females’ aged between 17 and 78 years (*M* = 28.90, *SD* = 9.44), whereas males’ age ranged between 19 and 78 years (*M* = 29.34, *SD* = 9.14). Such a wide age range was established to provide sufficient variance for examining the moderating role of relationship length on the association of partners’ EI, and to resolve the initial assortment vs. convergence issue. Relationship length, for those who reported it, ranged between six months (which was a required minimum length for being included in the study) and 40 years (*M* = 7.87, *SD* = 8.69).

### Measures

EI was assessed by TIE (Śmieja et al. 2014), a performance measure based on the ability model of EI. TIE comprises 24 tasks describing emotion-laden problems that require participants to indicate which emotion is most probable in the given situation, or to suggest the most appropriate action. Following the ability model of EI, TIE consists of four subscales (branches): Perception, Understanding, Facilitation, and Emotion Management. Correct answers are determined by expert samples: professional psychotherapists, trainers, and human resources specialists. The validation study showed that TIE is a reliable and valid test, suitable for both scientific research and individual assessment (Śmieja et al. 2014). Internal consistency of TIE is .88 (for the subscales, Cronbach’s alphas are: .70 (Perception), .69 (Understanding), .65 (Facilitation), .66 (Management). In line with the theoretical model of EI, the TIE scores share about 10 % of common variance with general intelligence test, and are independent from major personality dimensions.

## Results

To test the formulated hypotheses, we computed Pearson’s correlation coefficients between male and female partners’ EI branch and total scores. The results are presented in Table 1, accompanied by descriptive statistics and a dependent *t*-test, comparing average male and female scores. A rationale for using dependent *t*-test in case of matched-pairs investigations is provided by King and Minium (2003).

**Table 1:** Descriptive statistics, between-group mean comparisons, and between-partners Pearson’s correlation coefficients *N* = 382 couples

	Females		Males		t	g^†^	r
	M	SD	M	SD			
Perception	8.22	1.50	7.44	1.69	7.77***	.49	.23***
Understanding	7.65	1.41	6.98	1.51	7.03***	.46	.18***
Facilitation	7.17	1.34	6.73	1.48	4.81***	.32	.18***
Emotion Management	6.64	1.27	5.84	1.38	9.40***	.60	.22***
Total score EI	29.69	4.19	26.99	4.66	9.81***	.61	.27***

The dependent *t* tests are paired sample comparisons of female versus male means, *df* = 381. The *t*-tests were one-tailed, the r-Pearson correlations were two-tailed

^†^Hedges’ effect size measure

***p < .001

As in the previous studies based on ability measures of EI (e.g., Brackett et al. 2004; Brackett et al. 2006a, 2006b), females scored higher on each EI branch and total score. The effect sizes were moderate to high, ranging from .32 for Facilitation to .61 for total score.

The correlation coefficients revealed a systematic positive assortative mating effect, reaching a value of *r* = .27 for the EI total score. Because individuals -– on average - tend to be more similar to each other than dissimilar because of their shared cultural values, social desirability, and various response biases (see Klohnen and Mendelsohn 1998), it seems necessary to carefully evaluate the actual degree of similarity while applying dyadic design (Luo and Klohnen 2005). One of the most frequently applied methods is to calculate an average correlation for a number of randomly generated ‘pairs’ of females and males (using data from the actually analysed sample). In order to provide a comparison between the degree of actual couple similarity and the base-rate degree of similarity that exists in members of a population, we created ‘pseudo-couples’ by randomly pairing females and males from our sample. To obtain more reliable distribution of random couple similarity, we performed this procedure 10 times. Therefore, a maximum of 3820 random ‘couples’ were obtained. An average correlation for random couples was *r* = −.04, and differed significantly from the one obtained from actual couples (*p* < .001).

To verify which EI branches are related most strongly to partner’s EI, we conducted two multiple regression analyses. Female (in model 1) and male (in model 2) total EI scores were introduced as outcome variables, and their partner’s EI branch scores were introduced as predictors (see Table 2).

**Table 2:** Regression analyses predicting male and female general EI score with partner’s branch scores. *N* = 382 couples

Predictors	*B*	β	*p*	Model parameters
Model 1: Predicting female total EI score with male partners’ EI branch scores				
Perception	.30	.12	.05	*R* = .27
Understanding	.18	.07	.29	*R* ^2^ = .07
Facilitation	.14	.05	.44	adjusted *R* ^2^ = .06
Management	.34	.11	.05	*F*(4, 377) = 7.30, *p* < .001
Model 2: Predicting male total EI score with female partners’ EI branch scores				
Perception	.35	.11	.07	*R* = .27
Understanding	.22	.07	.22	*R* ^2^ = .07
Facilitation	.38	.11	.08	adjusted *R* ^2^ = .06
Management	.22	.06.	.33	*F*(4, 377) = 7.21, *p* < .001

Both models were significant, *F*(4, 337) = 7.30, *p* < .001, and *F*(4, 337) = 7.21, *p* < .001, and in both cases the explained amount of variance amounted to *R* ^*2*^ = .07 (.06 for adjusted *R* ^*2*^), and Cohen’s *f* ^*2*^ effect size indicator amounted to *f* ^*2*^ = .08.

In both cases, Perception branch turned out to be the strongest predictor of a partner’s total EI score, based on *β* coefficients. For females, the effect was equal to Facilitation in terms of size. For males, Emotional Management was also a significant predictor, whereas in women, there was a trend in predicting male EI score from Perception and Facilitation.

To verify whether the obtained effects originated in the initial assortment or the convergence, we applied the Continuous Parameter Estimation Model (CPEM; Gorsuch 2005), which estimates whether the correlation between two variables changes when a given third variable increases from low to high values. In our case, we checked whether the correlation between both partners’ EI changes when the relationship length grows. As only 185 of 382 couples reported relationship length – and in those who reported it a correlation with the average age of the couple was very high (*r* = .89, *p* < .0001) – we decided to treat the latter as an indicator of the former. The procedure consisted of the following steps: 1) All variables were standardized; 2) The product of male EI x female EI was formed; and 3) The correlation between the male EI x female EI product and age was determined. The correlation coefficient amounted to *r* = −.08, *p* = .11, which suggests that the assortative mating for EI is rather a consequence of the initial assortment. We replicated the CPEM analysis on the subsample of 185 couples that provided information on relationship length. The correlation coefficient was *r* = −.07, *p* = .38, confirming the effect obtained by using mean age as an indicator of relationship length.

As CPEM does not fully rule out the possibility of the confounding effects of age, we also applied partial correlation analysis, controlling for age. The correlations did not change significantly, with .27, *p* < .001 for EI total score (which is exactly the same as the zero-level correlations), .25, *p* < .001 for Perception, .18 *p* <. 001 for Understanding, .21 *p* < .001 for Facilitation, and .20, *p* < .001 for Management. Only the latter correlation was slightly lower when age was controlled, although Steiger’s Z analysis showed that the difference was not significant.

## Discussion

The current study sought to examine the degree of similarity between romantic partners in emotional intelligence. We assumed that people would select partners basing on a mutual ability to perceive, understand, manage and express emotions. According to our best knowledge, the present study is the first attempt to explore that aspect of human pairing using such a large sample. The results provide support for the assortative mating effect. The correlation coefficients between partners’ EI reached *r* = .27. As the strength of the correlation obtained in the present research did not depend on the length of the relationship and/or age, it is highly possible that initial mating, not the convergence process, is responsible for this effect. Since our sample was limited to students and university graduates, we suppose that the participants had rather similar social background (studying in a big city). Significant assortative mating effect found within such a homogenous sample (level of education is the major source of homogamy effects), suggests that the effect it is not the result of social homogamy, but a product of an active partner choice (Watson et al. 2004).

Beyond the positive assortment on the general EI level, we have also analysed to what extent specific emotional skills predict choosing a partner similar in EI. Interestingly, branch-level predictors of partner’s EI differed for men and women. For males, two groups of skills were predictive of their female partner’s EI: perception of emotion and management of emotion. Those men who were more efficient in perceiving emotions and regulating them attracted more similar women. Accurate emotion perception appears to be a helpful skill in tracking partners who display similar level of emotional skills. For someone apt at decoding emotional signals it seems easier to find a “matching” person. The role of management of emotions in males mating strategy appears more speculative. One of the possible explanations takes into account a particularly pronounced physical attractiveness related motivations present in males’ partner selection (Buss 1989). To limit the dominance of these basic evolutionary drives related to fertility cues, and consider some other, more sophisticated criteria for partner selection, including EI level, man need the ability to control emotions. Probably, efficient regulation of primal drives and affects allows him to focus on other important aspects of a potential partner, not only her physical attractiveness. On the other hand, we cannot forget that the Management branch also relates to regulating others’ emotions. Thus, males scoring high on this branch may influence females’ emotions more effectively and may be more attractive (or more ‘gratifying’) to females focused on satisfying their own emotional needs (i.e., for highly emotionally intelligent females).

In females, management of emotions did not predict finding similar partner, however the second marginally significant predictor beside Perception was the Facilitation branch. As we noted in the introductory part of this paper, females’ mate selection criteria are different from males’ criteria as more focused around resource acquisition (Buss 1989). To properly assess a male’s mate value, a detailed cognitive analysis of his current and potential resources is necessary. Facilitation branch refers to those aspects of EI that are related to facilitation of cognitive processes, including rational decision making. Selecting a valuable and/or truly promising partner may thus be supported directly by this ability. Since high level of EI is a predictor of an individual’s potential to gain resources (e.g., Lopes et al. 2006), including this criterion by females facing a mate-choice decision may indirectly result from elevated levels of emotion facilitation abilities. In conclusion, our results show that for positive assortative mating, accurate perception of emotions might be important for both sexes, while skillful management of emotions seems more crucial for men, and efficient facilitation of emotions – more essential for women. Interestingly, understanding of emotions did not show its significance in predicting partner’s EI. This might be because this ability, including understanding emotional language and dynamics, is less related to direct interpersonal interactions, and thus its role in mating strategy may be negligible. Although these explanations of the obtained effects are all post-hoc and speculative, in the light of major findings of evolutionary psychology, they seem quite plausible.

The similarity of EI in couples suggests that this characteristic could be one of the important factors in mating decisions. Taking into account how important the skills constituting EI might be for the well-being of a relationship, it seems puzzling why the effect is not even stronger. Presumably, the reason stems from the difficulty of assessing emotional abilities.

People learn their own mate’s value by comparing themselves to other members of a social group. During repeated experiences of acceptance and rejection in interactions with peers, especially in adolescence, we build our internal index of personal value (Todd and Miller 1999). Such self-assessment processes can be regarded as a mate value sociometer (Leary et al. 1995; Kirkpatrick and Ellis 2001), which serves the function of guiding adaptive relationship choices.

Self-perceived value as a mate plays an important role in human preferences of mate selection as it translates directly to what people expect of their prospective mates (Edlund and Sagarin 2010). To calibrate such mating sociometer adaptively, individuals should assess their own features and the features of potential partners accurately. It might be difficult in the case of EI because appraising emotional abilities is not an easy task. During their lifetime, people receive little explicit feedback about their emotional abilities in comparison to other mental skills, which results in insufficient knowledge about that aspect of self. Numerous studies show that people are not able to assess their EI level precisely (correlations between self-reports and ability measures are low or insignificant), and usually exhibit a strong self-enhancement bias in estimating their emotional skills (Brackett et al. 2006a, 2006b; Brackett and Mayer 2003; Brannick et al. 2009; Zeidner et al. 2005). Therefore, self-assessment of EI (and presumably assessment of others’ EI) seems far from accurate, and as such – it is not a perfect basis for assortative mating. Most likely, partners would be able to match their levels of EI more precisely if they were able to estimate them more precisely. This interpretation is to some degree supported by the results of a study by Smith et al. (2008), who found an assortative mating effect only for the perceptions of partner’s EI, but not for a partner’s own reports of EI.

### Strengths, Limitations and Future Studies

The current research addresses a significant gap in the literature concerning assortative mating for ability emotional intelligence. It is the first study engaging such a large sample not limited to students, but people of different ages and various relationship lengths, which allows tracing potential convergence effect. However, the present study has several limitations. The first comes from the typical disadvantage of a cross-sectional design, attributable to inability of determining a casual interference. The second is the amount of missing data (~48 %) in couples’ reports of relationship length. Based on the correlation between age and relationship length, we assumed that the former could be used as a proxy for the latter. However, using the present data we cannot determine whether the omissions in length were random or not. If they were somehow related to relationship length (i.e., couples with either shorter or longer relationships systematically omitted that information), our conclusion regarding convergence may be inaccurate. Further, because as we mentioned in the introductory section, the TIE scores are moderately *g*-loaded (Śmieja et al. 2014), future studies should control for IQ to determine to what extent the assortative mating effect for EI results from the effect of assortative mating for general intelligence (Bouchard and McGue 1981). In addition, other dimensions for which assortative mating effects were demonstrated (e.g., personality, attitudes, and values) could have been included as covariates, although that seems less important than including IQ, as ability EI shares much smaller portion of variance with these areas (e.g., Mayer et al. 2008).

Because the very premise of assortative mating is that it should enhance relationship quality, testing this hypothesis is actually a common practice in studies of assortative mating effects (e.g., Luo and Klohnen 2005; Watson et al. 2004). A lack of such relationship outcome variables is certainly a limitation of the present study, and future research should include such measures (e.g., relationship satisfaction). Following this line of reasoning, it could be also illuminative to include sexuality-related variables (e.g., sexual satisfaction, preferences, attitudes or behaviors) in the next studies in order to test their potential mediating role in the assortment processes. Future studies could also compare assortative mating for EI using other than TIE measures of emotional intelligence. We hope that all these variables and directions will be examined in the future, because the studies that address assortative mating and partner selection seem inherently important.
